# Quality of primary health care for chronic diseases in low-resource settings: Evidence from a comprehensive study in rural China

**DOI:** 10.1371/journal.pone.0304294

**Published:** 2024-07-25

**Authors:** Mingyue Li, Xiaotian Zhang, Haoqing Tang, Huixian Zheng, Ren Long, Xiaoran Cheng, Haozhe Cheng, Jiajia Dong, Xiaohui Wang, Xiaoyan Zhang, Pascal Geldsetzer, Xiaoyun Liu

**Affiliations:** 1 Department of Health Policy and Management, School of Public Health, Peking University, Beijing, China; 2 China Center for Health Development Studies, Peking University, Beijing, China; 3 Division of Primary Care and Population Health, Department of Medicine, Stanford University, Stanford, California, United States of America; 4 School of Health Policy & Management, Nanjing Medical University, Nanjing, China; 5 Department of Cardiology, Fuwai Central China Cardiovascular Hospital, Zhengzhou, China; 6 Department of Social and Behavioral Sciences, School of Global Public Health, New York University, New York, New York, United States of America; 7 Chan Zuckerberg Biohub, San Francisco, San Francisco, California, United States of America; National center for chronic and non-communicable diesease prevention and control, CHINA

## Abstract

**Background:**

There is a paucity of evidence regarding the definition of the quality of primary health care (PHC) in China. This study aims to evaluate the PHC quality for chronic diseases in rural areas based on a modified conceptual framework tailored to the context of rural China.

**Methods:**

This comprehensive study, involving a patient survey, a provider survey and chart abstraction, and second-hand registered data, was set in three low-resource counties in rural China from 2021 to 2022. Rural patients with hypertension or type 2 diabetes, and health care workers providing care on hypertension or diabetes were involved. The modified PHC quality framework encompasses three core domains: a competent PHC system (comprehensiveness, accessibility, continuity, and coordination), effective clinical care (assessment, diagnosis, treatment, disease management, and provider competence), and positive user experience (information sharing, shared decision-making, respect for patient’s preferences, and family-centeredness). Standardized PHC quality score was generated by arithmetic means or Rasch models of Item Response Theory.

**Results:**

This study included 1355 patients, 333 health care providers and 2203 medical records. Ranging from 0 (the worst) to 1 (the best), the average quality score for the PHC system was 0.718, with 0.887 for comprehensiveness, 0.781 for accessibility, 0.489 for continuity, and 0.714 for coordination. For clinical care, average quality was 0.773 for disease assessment, 0.768 for diagnosis, 0.677 for treatment, 0.777 for disease management, and 0.314 for provider competence. The average quality for user experience was 0.727, with 0.933 for information sharing, 0.657 for shared decision-making, 0.936 for respect for patients’ preferences, and 0.382 for family-centeredness. The differences in quality among population subgroups, although statistically significant, were small.

**Conclusion:**

The PHC quality in rural China has shown strengths and limitations. We identified large gaps in continuity of care, treatment, provider competence, family-centeredness, and shared decision-making. Policymakers should invest more effort in addressing these gaps to improve PHC quality.

## Introduction

Non-communicable diseases have become the major contributors to death and disability worldwide. Globally in 2019, 75% of the disability-adjusted life-years lost (DALYs) were because of NCDs [1]. Primary health care (PHC) is the cornerstone of health systems to tackle the challenge of NCDs [2]. The 2018 Declaration of Astana reaffirmed the fundamental role of PHC in enhancing people’s physical and mental health as well as social well-being [3]. It is universally acknowledged that PHC creates the foundation to achieve universal health coverage (UHC) and the health-related Sustainable Development Goals (SDGs). The underlying assumption is that PHC has adequate quality to optimize health. However, this assumption is not always valid, especially in low-income and middle-income regions. Studies have shown that widespread gaps still exist in PHC quality [4, 5], such as undertreatment and poor management of non-communicable diseases [4], overuse of antibiotics [6] and low coordination of services [7]. Poor PHC quality can jeopardize trust, leading people to bypass PHC and seek expensive specialty care.

PHC quality is strongly correlated to the services provided. Due to population ageing and shifts in lifestyles, the burden of hypertension-related diseases and diabetes mellitus has rapidly increased. The annual deaths associated with hypertension have increased from 97.9 to 106.3 per 100,000 population from 1990 to 2015 [8]. 529 million people are living with diabetes worldwide in 2021, and the DALYs count has increased by 189.8% since 1990 [9]. The growing chronic disease burden has resulted in changes in PHC delivery and health services. Multiple national strategies have been adopted to strengthen PHC service delivery [10], which will affect how to define and measure quality. For example, the transition of care from hospitals to community settings for chronic diseases (changes in delivery) and the shift towards integrating prevention services, such as early screening for hypertension, into routine clinical care (changes in services) will impact PHC quality.

In theory, PHC is well positioned to respond to chronic diseases if it promotes first contact and a broad range of basic care to all people [2]. However, PHC in rural areas is often understaffed, placing healthcare workers under strain to deal with the growing disease burden [11]. Constrained resources and insufficient external support also undermine the job performance and productivity of healthcare workers [12]. PHC workers in rural areas often have low medical qualifications, which subsequently leads to low-quality services [13, 14]. However, studies evaluating PHC quality in rural areas have mainly relied on standardized patients, vignettes, or subjective measurements of patient experiences [15, 16]. These methods primarily reflect the quality of episodic care. The unique advantages of PHC in responding to chronic diseases, by comprehensively and continuously meeting most basic health care needs at low costs, are overlooked in these methods.

Most research about PHC quality has been conducted in high-income countries, while the respective metrics are frequently unavailable in rural areas. Global initiatives have been actively advancing studies on PHC quality in low-income and middle-income countries [5, 17, 18]. Our previous systematic review synthesized the progress in this field and summarized the definitions and frameworks of PHC quality [19]. These frameworks have established a solid knowledge base for understanding, evaluating and improving PHC quality. Notably, the mapping of PHC indicators to the High-Quality Health System (HQSS) framework [5] and the Primary Health Care Performance Initiative (PHCPI) framework are two major efforts [20]. These two frameworks shed light on the “black box” (the process of care) of PHC quality, rather than treating quality as one single element. Studies have applied these two frameworks to evaluate PHC quality in low-income and middle-income countries [5, 17].

PHC quality is closely associated with contextual factors, including disparities in health systems, economic power, and political backgrounds, alongside the spectrum and burden of diseases [21, 22]. Applying a uniform PHC quality framework without accounting for these variations may lead to incongruities. For instance, the cooperation between clinics and secondary hospitals is an essential component of care coordination. However, in areas where the health system is not fully developed, there might be no established system for referrals. Consequently, researchers typically modify the general framework to suit the local context before implementation. For example, Rezapour et al. developed an Iranian PHC quality framework based on the WHO framework and other frameworks, ensuring alignment with Iran’s health system [23].

This study contributes to the existing literature along the following dimensions. We aim to evaluate the PHC quality for chronic diseases in rural China using a modified framework that has been enhanced for greater applicability and relevance, drawing from recent global frameworks. Hypertension and diabetes were used as tracer conditions. In sum, our findings add knowledge to understanding and enhancing PHC quality for chronic diseases, not only in rural China but also in other low-income and middle-income countries that experience transformative phases in their health systems.

## Methods

### The settings

This study follows the STROBE checklist for observational cross-sectional studies. This study was approved by the Institute Review Board (IRB) of Peking University Health Science Center (IRB00001052-22155). Written informed consent was obtained from all participants and health organizations prior to questionnaire. This cross-sectional study was conducted in three counties of three provinces in western and central China, Hubei, Henan, and Shanxi Province. The socioeconomic status and the health resources in the three counties were all below the national average (Table A3 in S1 Appendix). Primary care is predominantly delivered by village clinics, township health centres and county hospitals in rural China. Most village clinics are privately owned, with some being affiliated with township health centres. Township health centers are public institutions and receive full government subsidies, while county hospitals, also public institutions, are partially subsidized by government funding [24]. County hospitals traditionally provided secondary care, but as more people seek outpatient care as first-contact care, county hospitals are now playing the role of PHC [25].

China’s rural PHC system is undergoing reforms, with the most notable being the medical alliance system that started in 2015 [26]. County hospitals and PHC facilities build networks of care, with or without unified administrative management. County hospitals and PHC facilities are assigned clear roles and responsibilities. The reform aims to build a patient-centred dual referral pathway alongside the care continuum [10]. In the three project counties, county A in Shanxi province has built one medical alliance including all PHC facilities with a unified administrative system (administration, personnel, business, funds, medical devices, and performance evaluation etc.). County B in Henan province and County C in Hubei province have both built two medical alliances within each county, in collaboration with several PHC facilities, each led by a county hospital.

PHC facilities have transitioned from solely focusing on clinical care to expanding to public health services. To respond to the challenges of chronic diseases, China launched the National Basic Public Health Service Program in 2009. PHC facilities started to deliver a comprehensive package of public health services to all residents free of charge, encompassing vaccination, health examinations, screening, health management and health education. A key component of the service package is the registration and management of patients with hypertension or type 2 diabetes [27].

The concept of participant involvement translated to the study design and execution phases of the research. The development of the research question was based on public concerns. Patients and doctors were recruited for the study, and the study design was explained in detail. We worked with investigators from communities, and they reviewed the questionnaires first and gave feedback. We disseminated the study results through public policy briefs.

### Conceptual framework of PHC quality

As a primary source of information, we drew on the HQSS framework. The HQSS includes three domains: 1) competent systems (safety, prevention and detection, continuity and integration, population health management, and timely action), 2) evidence-based care (technical quality indices for key PHC services), 3) positive user experience (patient focus, clear communication) [5, 28]. As a second source, we use the PHCPI framework, a collaborative effort involving the Bill & Melinda Gates Foundation, the World Bank, WHO, and other partners [17, 20]. PHCPI offers a comprehensive evaluation of the overall performance of the PHC system, with a specific emphasis on service delivery. Within the service delivery section of PHCPI, key dimensions include population health management, facility organization and management, access, availability of effective PHC services, and high-quality PHC. PHCPI emphasizes essential structural contents tailored for low-income and middle-income countries, encompassing information systems and facility management. Our research aligns with the quality focus delineated in the service delivery section of PHCPI, with a particular emphasis on the distinctive functions of PHC.

**Fig 1:**
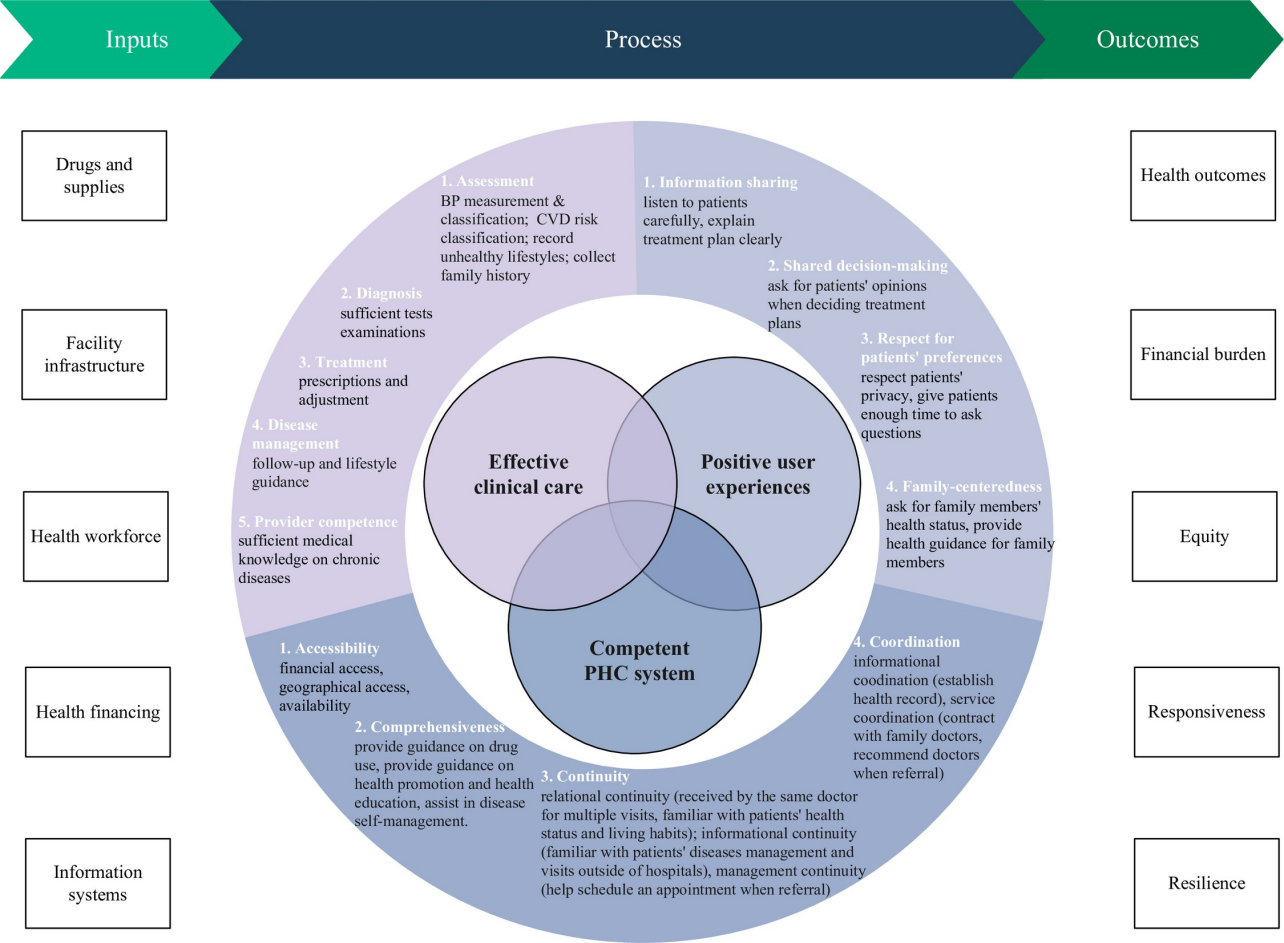
The modified conceptual framework for the primary health care quality for chronic diseases in rural China.

Drawing on HQSS and PHCPI, we updated the conceptual framework for evaluating the PHC quality for chronic diseases in rural China. High-quality PHC for chronic diseases should underpin three core domains: a competent PHC system, effective clinical care, and positive user experience (Fig 1). We added the provider competence from the PHCPI to the clinical care sub-domain because providers in rural China have been long criticized for having insufficient ability and medical knowledge [4]. We included disease management as a sub-domain due to our focus on chronic diseases and the increased time spent on disease management by PHC providers after the National Basic Public Health Service Program implementation. We simplified sub-domains for user experiences compared with the HQSS, because some of them are not suitable for rural China’s context. For example, discrimination or social stigma may be associated with certain chronic diseases (such as mental illness) in rural China [29], but it is not a primary concern for hypertension or diabetes.

While we recognized that inputs and outcomes were also highly important, this study identified quality more as a concept of process. The building blocks of health system inputs, including drugs and supplies, facility infrastructure, health workforce, health financing, and information systems, were fundamental in building a competent PHC. PHC without positive outcomes, including health outcomes, financial burden, equity, responsiveness, and resilience, is ineffective.

The definitions for quality domains and sub-domains are presented in Table 1. Quality for PHC system focused on the unique functions of PHC compared to specialty care. Accessibility, comprehensiveness, continuity, and coordination were universally recognized as essential to achieving an effective PHC system. Quality for clinical care focused on the continuum of patient journey for chronic diseases which refers to the degree to which clinical processes conform to existing evidence and/or widely accepted standards [5, 30]. For chronic diseases, the continuum of care covers disease assessment, diagnosis, treatment and disease management, and provider competence that ensures an effective care continuum. Quality for user experience refers to information sharing, shared decision-making, respect for patient preferences and family-centeredness [31].

**Table 1:** Definitions and data sources for quality domains and subdomains.

Quality domains	Sub-domains	Definitions	Data sources
PHC system	Accessibility	The ease with which people can access and utilize healthcare services within a timeframe that aligns with their personal health needs [30, 32]. This includes geographic accessibility, financial accessibility, and availability.	Patient questionnaire survey
	Comprehensiveness	The extent to which PHC can meet the comprehensive health care needs of patients. This includes basic public health services such as preventive care and health education, as well as diagnosis and treatment of common diseases, referrals, and health management services [32].	Patient questionnaire survey
	Continuity^a^	The degree to which a patient’s series of discrete healthcare utilization behaviours are interconnected and consistent. This includes management continuity, informational continuity, and relational continuity [33].	Patient questionnaire survey
	Coordination^b^	The degree to which health care services are coordinated and integrated to meet individual needs. This includes service coordination and informational coordination [31, 34].	Patient questionnaire survey
Clinical care	Assessment	The ability to appropriately inquire about medical history to collect relevant clinical information from patients, and risk classification based on the patient’s health status.	Chart abstraction of inpatient medical records
	Diagnosis	The ability to make decisions based on sufficient evidence, including adequate laboratory tests, examinations and so on.	Chart abstraction of inpatient medical records
	Treatment	The ability to prescribe appropriate medications without overuse or underuse, timely referrals, or recommendations for hospitalization.	Chart abstraction of inpatient medical records
	Disease management	Based on collecting personal health information, anticipating the risk of developing a certain chronic disease in individuals within a defined period in the future. Based on the risk prediction, formulate personalized prevention plans with regular follow-ups and effectiveness assessment [35]. For chronic diseases in rural China, refers to adequate frequency of follow-ups.	Patient questionnaire survey; National register data of basic public health services
	Provider competence	The ability of health care providers to use their knowledge, skills, and judgement to provide care effectively. Chronic diseases in rural China refer to healthcare workers having adequate knowledge of chronic diseases.	Provider questionnaire survey
User experiences	Shared decision-making	Patients and providers make health decisions together. For chronic diseases in rural China, refers to providers including patients’ opinions when formulating a treatment plan.	Patient questionnaire survey
	Family-centeredness	The degree to which providers identify the family as a social determinant of health, thus developing and implementing care plans focusing that include the family [36]. Chronic diseases in rural China, refer to inquiring about family history and the health of family members.	Patient questionnaire survey
	Information Sharing	The degree to which providers share complete and unbiased information with patients and families. For chronic diseases in rural China, refers to offering patients access to their medical records and files.	Patient questionnaire survey
	Respect for patient preferences	The degree providers value the choices, desires, and values of patients regarding their health. For chronic diseases in rural China, refers to whether patient privacy is respected and if patients are given sufficient time to ask questions.	Patient questionnaire survey

^a^There are certain overlaps between the concepts of management continuity and coordination, and some studies do not distinguish the two concepts [37]. In this study, management continuity primarily focuses on the management and follow-up of chronic diseases, emphasizing the behaviours of healthcare workers.

^b^Coordination primarily focuses on family doctor contracting, health records, and information systems, emphasizing the organization, construction, and management of health resources within and across health facilities.

### Data source and participants

Multiple data sources including patient questionnaire surveys, provider questionnaire surveys, medical records of health facilities, and register data from governments were used to evaluate PHC quality (Table 1). Hubei, Henan, and Shanxi provinces that are located in less-developed western and central areas were selected based on willingness to cooperate and logistic considerations, as higher local willingness would suggest a higher level of healthcare worker engagement and better data quality. Three low-resource counties and nine towns were selected. The administrative structure in China is strictly hierarchical, consisting of a province/municipality at the first level, and a county, a **township**, or a village in a rural area.

We conducted a patient questionnaire survey to collect data on the PHC system and user experiences. The questionnaire collected information on basic household and personal characteristics, outpatient visits and inpatient visits. We used a two-stage stratified cluster random sampling in patient survey. The strata were regions (county A in Hubei, county B in Henan, and county C in Shanxi) and villages. In the first stage, a random sample of 9 towns was selected whereby 3 towns were selected in each county. Using a significance level of 0.05 with 3% absolute precision, we estimated that a sample size roughly 1000 would be necessary. In the second stage, we determined the quota of 900 hypertensive patients and 450 diabetic patients whereas 300 hypertensive patients and 150 diabetic patients in each county. Registration dates before 07/30/2022 were included. Because patients were not randomly registered but based on the dates of disease detection, and newly detected patients typically exhibit milder symptoms, we used a systematic random sampling of patients’ villages instead of selecting individual patients. This cluster approach enabled us to include patients with diverse levels of severity. All individuals with hypertension or diabetes living in the selected villages were recruited between 07/10/2022 and 07/27/2022. In the field survey, 1357 patient questionnaires were included, two were excluded due to a lack of significant information on quality-related questions. The questionnaire can be found in the S1 Appendix.

We also conducted a provider survey to collect information on provider competence. The questionnaire collected information on basic personal characteristics, service provision, job satisfaction and vignettes of patient scenarios with varied severity of hypertension or diabetes. We used a cluster sampling approach, and all the healthcare workers providing care for hypertension or diabetes (village doctors, general practitioners (GP), public health doctors, internists) in the three county hospitals (n = 50), nine towns (n = 76) and all villages (n = 199) were recruited between 07/11/2022 and 07/27/2022. The response rate for providers was 94.0% in county hospitals, 84.2% in township health centres and 89.9% in village clinics. The questionnaire can be found in the S1 Appendix.

We reviewed and abstracted medical records using pre-determined checklists to collect data on disease assessment, diagnosis and treatment, a method known as chart abstraction, which is widely used to evaluate quality of care [16, 38]. We collected detailed information on clinical processes, such as whether performing glycated haemoglobin tests for diabetic patients or classifying patients based on blood pressure levels. The detailed checklists can be found in the S1 Appendix. Patients with primary discharge diagnoses of hypertension, diabetes, or related complications from 01/01/2020 to 06/30/2022 were included. Patients with another primary diagnosis (i.e. poisoning) but with a history of hypertension, diabetes or related complications were excluded. Data were primarily abstracted from inpatient medical records, including both paper and electronic records. Other electronic health systems were also used for supplementary information, including the Hospital Information System (HIS) system, the imaging system, and the laboratory system. Clinical experts with pre-trained investigators reviewed medical records, abstracted information, and filled up the checklists. All checklists were double-checked at the end of the day by investigators and clinical experts. The medical records were accessed between 07/11/2023 and 07/27/2023 for research purposes, and no identifiable individual information was collected.

Health records and follow-up visits for chronic diseases are registered in the national basic public health services system. The anonymized data on hypertension/diabetes follow-up visits were exported from the registry. The data contained time and blood pressure/glycated haemoglobin measurement for each follow-up visit. Detailed descriptions of indicators for each domain and sub-domain are presented in the S1 Appendix.

### Calculation of quality scores

Five quality scores were calculated for three domains of PHC quality at the patient or provider level. Due to various data sources, clinical care is reflected by separate sub-domain scores. The score of provider competence was calculated at the provider level, while all other scores were calculated at the patient level. The list of indicators for each domain and sub-domain is reported in the S1 Appendix.

All PHC quality domains were composed of binary (ie, 0 or 1) indicators and were then summarized into domain scores or sub-domain scores ranging from 0 (lowest) to 1 (highest). Scores above 0.7 were considered favourable or optimal. Different strategies were used for summarizing quality scores due to data structure and sources: 1) for the quality score of PHC system, disease management and user experience, the arithmetic mean for all the indicators in the respective domain/sub-domain was calculated; 2) for the quality score of clinical care and provider competence, Item Response Theory (IRT) models were used. IRT has been widely used in educational and psychological testing for studying the relationship between participants’ latent characteristics (such as competence, and cognition) and their response to a set of questions. IRT has also been used in studies on PHC quality. For example, Das et al. also used IRT to analyze PHC quality in India in 2004 [39]. We used the Rasch model (also referred to as one parameter IRT model) to calculate quality scores for clinical care and provider competence. For the Rasch model (1), P is the probability of person j’s correct response to question i, and *θ* is the provider competence. The hypothesis was that questions vary only in difficulty (b_i_). The Rasch model can be transformed into (2), i.e. a typical logistic model, that can be estimated by the maximum likelihood method:

$$\text{P}\left(Y_{ij}=1|\theta_{j}\right)=\frac{\exp\left(\theta -b_{i}\right)}{1+\exp\left(\theta -b_{i}\right)},\theta_{j}∼\text{N}(0,1)$$ (1)

$$\text{Hence},\ln\;\left(\frac{\text{P}\left(Y_{ij}=1|\theta_{j}\right)}{1-\text{P}\left(Y_{ij}=1|\theta_{j}\right)}\right)=\theta_{j}-b_{i}$$ (2)

### Statistical analysis

We reported the levels of quality scores for each quality domain and sub-domain and then reported the distribution of quality scores by socioeconomic characteristics. We assessed gender (female, male), age (<50, 50–60, 60–70, ≥70), marriage (unmarried, married), education (≤primary school, middle school, ≥high school), household income group (in five quantiles), and medical insurance (Urban Employee Basic Medical Insurance (URBMI), Urban Employee Basic Medical Insurance (URBMI), New Rural Cooperative Medical Scheme (NCMS), Urban and Rural Resident Basic Medical Insurance (URRBMI)). We also conducted a series of sensitivity analyses to test how robust our findings were to modelling assumptions. Cronbach’s alpha was used to examine the reliability of the summative score (S1 Appendix). Since no summative score was built for the clinical care domain, we used multivariable linear regression models to further examine the association between provider characteristics and provider competence. As predictors, we assessed working places, gender, education, having permanent posts, having passed the medical licensing exam, job title, administrative roles, and job satisfaction. No sampling weights were used, and standard errors were clustered at the level of regions (towns). Missing data (unanswered questions) were imputed using multiple chained equations. P<0.05 was considered as statistically significant. All statistical analyses were conducted in Stata V.17.0 (Stata Corp LP).

## Results

### Characteristics of the study sample

In total, this study included 1355 patients, 333 providers and 2203 medical records (Table 2). Two patient questionnaires were excluded due to a significant missing information of quality-related questions. No provider questionnaires were excluded. No medical record abstractions were excluded. Patients in the patient survey were on average 65.8 years old, and 20.4% of the patients had a comorbidity of hypertension and diabetes. Providers came primarily from village clinics (53.7%). 77.9% of the inpatient medical records were from county hospitals. The average age for inpatients was 64.3 years old. This study primarily focused on patients from low-resource rural areas, which may not be comparable to the nationally representative surveys. Patients in our study comprised more women (62.4%) and were older than national representative samples.

**Table 2:** Characteristics of the study sample.

	Patients	Providers	Medical records
Sample size (N)	1355	333	2203
Regions, N (column %)			
Hubei	420 (31.0)	132 (39.6)	794 (36.0)
Henan	466 (34.4)	143 (42.9)	726 (33.0)
Shanxi	469 (34.6)	58 (17.4)	683 (31.0)
Age, Mean (SD)	65.8 (9.4)	41.3 (10.5)	64.3 (12.2)
Gender, N (column %)			
Female	845 (62.4)	162 (48.6%)	1085 (49.3%)
Male	510 (37.6)	171 (51.4%)	1118 (50.7%)
Health facilities, N (column %)			
Village clinics	NA	179 (53.7)	NA
Township Health Centers	NA	107 (32.1)	487 (22.1)
County hospitals	NA	47 (14.1)	1716 (77.9)
Survey year	2022	2022	2021–2022
Diseases, N (column %)			
Hypertension	901 (66.5)	NA^a^	850 (38.6)
Diabetes	178 (13.1)	NA	792 (36.0)
Comorbidity^b^	276 (20.4)	NA	561 (25.5)

^a^NA, not applicable. Village clinics in China do not provide inpatient services.

^b^Comorbidity refers to a dual diagnosis of hypertension and diabetes.

### Levels of PHC quality

**Fig 2:**
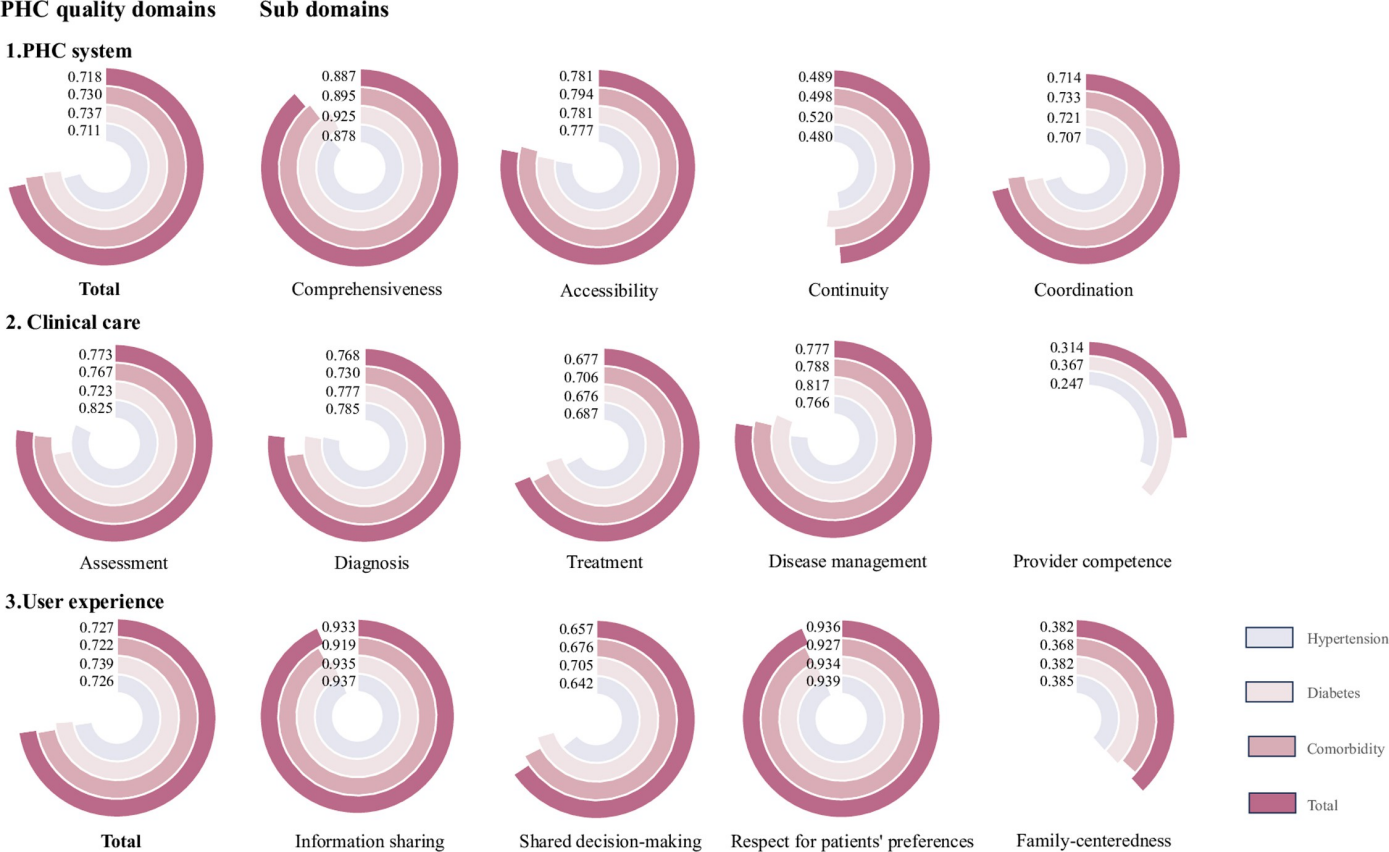
Average quality domain and sub-domain score of PHC for hypertension, diabetes, and comorbidity. For quality score, 0 represents the lowest quality, and 1 represents the highest quality. Clinical care score was displayed by sub-domains due to disparate data sources including chart abstraction, patient survey, provider survey and national registry data. Comorbidity refers to a dual diagnosis of hypertension and diabetes.

Fig 2 shows the PHC quality score for different domains and sub-domains. The overall PHC system score was 0.718 on average, and the user experience total score was 0.727 on average. Within the PHC system quality domain, comprehensiveness received the highest score (0.887), while continuity scored the lowest (0.489).

Clinical care score was displayed by sub-domains due to disparate data sources. Assessment (0.773), diagnosis (0.768), and disease management (0.777) demonstrated similar and favourable scores (scoring above 0.7 out of 1), whereas provider competence scored lower (0.314).

Within the user experience quality domain, information sharing (0.933) and respect for patients’ preferences (0.936) scored higher compared to shared decision-making (0.657) and family-centeredness (0.382).

The quality scores for diabetes were generally higher (0.737 for diabetes vs 0.711 for hypertension in the PHC system, 0.739 for diabetes vs 0.726 for hypertension in user experience), but the variations in the quality scores across diabetes, hypertension, and comorbidity were small.

### Distribution of PHC quality

**Fig 3:**
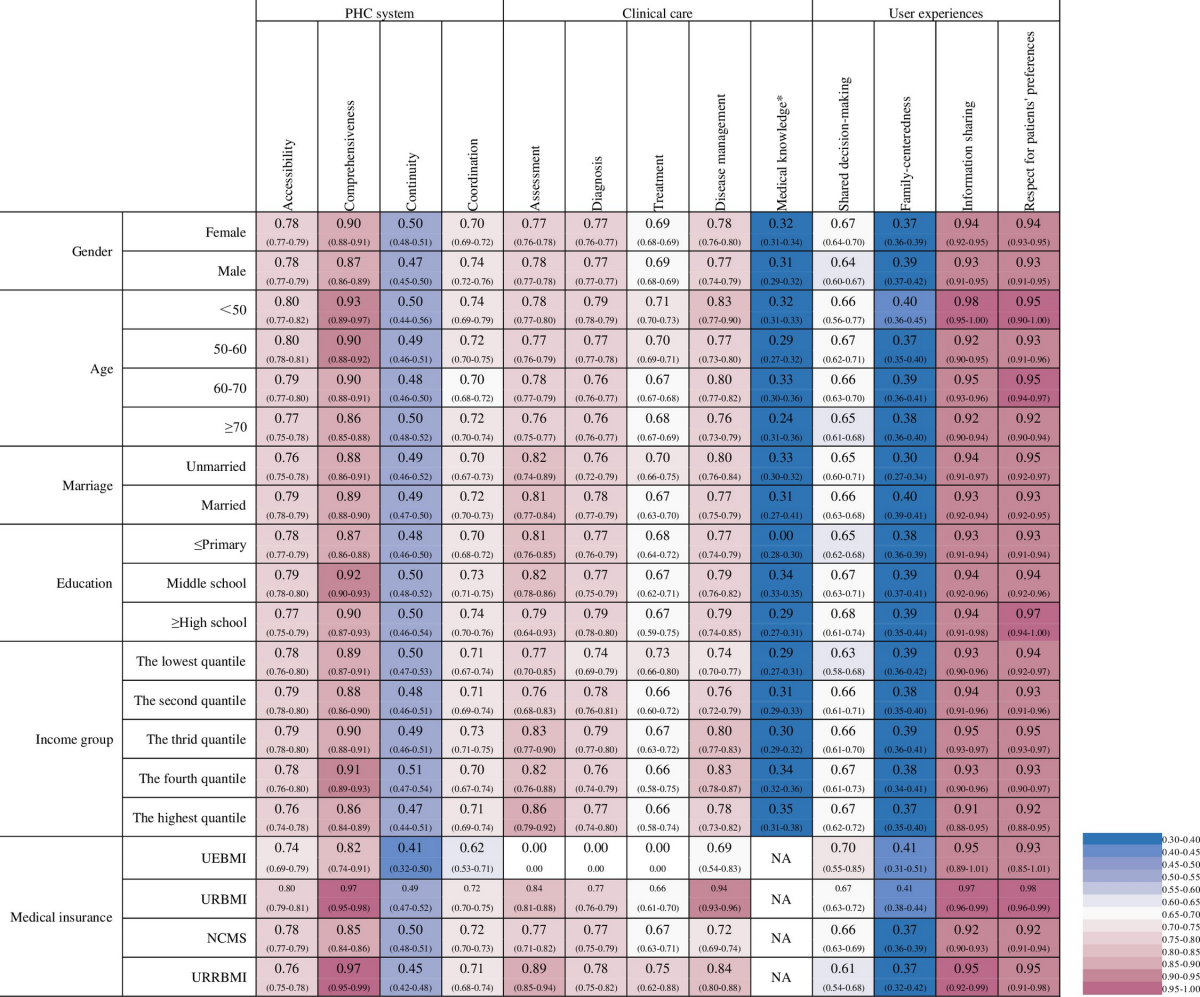
The distribution of average PHC quality score by socioeconomic characteristics (95% CI). The distribution of quality scores for assessment, diagnoses, and treatment in marriage, education, income group, and medical insurance were based on matched data between chart abstraction and patient survey; Quality scores for medical knowledge are at the provider level rather than the patient level. NA, is not applicable, meaning no observations in that category. UEBMI, Urban Employee Basic Medical Insurance. URBMI, Urban Resident Basic Medical Insurance. NCMS, New Rural Cooperative Medical Scheme. URRBMI, Urban and Rural Resident Basic Medical Insurance. Comorbidity refers to a dual diagnosis of hypertension and diabetes.

Fig 3 presents the PHC quality score and 95% confidence intervals by socioeconomic status, and Fig A1 in S1 Appendix presents the results of statistical tests (Fig A1 in S1 Appendix). The scores for comprehensiveness of care were higher for women (0.90 vs 0.87, P = 0.033), while the opposite was observed for scores in coordination (0.70 vs 0.74, P = 0.007). The younger patients (<50) showed higher quality scores in most quality dimensions compared to the elder groups (≥70), but similar scores in continuity (0.50 vs 0.50). Married patients revealed higher scores in accessibility (0.79 vs 0.76, P<0.001) and family-centeredness (0.40 vs 0.30, P<0.001).

The scores for comprehensiveness of care were higher in patients with higher education levels (high school) compared to those with primary school (0.90 vs 0.87, P<0.001), but lower compared to middle school (0.90 vs 0.92, P<0.001). Lower income groups showed lower quality scores in diagnosis (0.74 for the lowest income group vs 0.77 for the highest income group, P = 0.045), disease management (0.74 vs 0.78, P = 0.002), but higher scores in accessibility (0.78 vs 0.76, P<0.001), comprehensiveness (0.89 vs 0.86, P = 0.003) and other dimensions in quality of user experience.

We conducted reliability checks for quality domains of the PHC system, user experience and provider competence, and the respective Cronbach’s α were 0.583 and 0.711 (S1 Appendix). As for clinical care, we followed Chinese clinical guidelines for hypertension and diabetes, National Basic Public Health Service Standards, and consulted expert opinions. We conducted sensitivity analyses to examine the associations between several factors and provider competence. Compared with providers at village clinics, providers at township health centres and county hospitals had significantly better competence. Providers at county hospitals had 26% higher competence than those at village clinics (P = 0.005). This association was consistent across models (Table 3). Providers with permanent posts had 10% better competence than those without (P = 0.039). Education was not statistically significant because it has been largely explained by working places ‐ 75% of providers at village clinics had a high school or below education while all providers at the county hospital and 92% of providers at township health centres had a college or above degree.

**Table 3:** The associations between provider’s characteristics and provider competence^a^.

Variables	Model 1			Model 2			Model 3		
	Coef.	95% CI	*P value*	Coef.	95% CI	*P value*	Coef.	95% CI	*P value*
Working places (ref. = village clinics)									
Township Health Centers	0.22	(0.05 to 0.38)	0.016	0.21	(0.03 to 0.38)	0.024	0.20	(0.01 to 0.38)	0.040
County hospitals	0.23	(0.12 to 0.35)	0.001	0.27	(0.13 to 0.40)	0.001	0.26	(0.10 to 0.42)	0.005
Male (ref. = female)				0.01	(-0.07 to 0.08)	0.880	-0.01	(-0.08 to 0.05)	0.633
Education (ref. = college or above)									
High school or below				-0.01	(-0.10 to 0.09)	0.910	0.01	(-0.07 to 0.10)	0.709
Permanent posts (ref. = none)							0.10	(0.06 to 0.18)	0.039
Having passed the medical licensing exam (ref. = none)							0.03	(-0.02 to 0.08)	0.193
Job title (ref. = none)							-0.06	(-0.15 to 0.02)	0.140
Administrative roles (ref. = none)							-0.01	(-0.08 to 0.06)	0.773
Overall job satisfaction (ref. = not satisfied)							0.08	(-0.01 to 0.17)	0.069

^a^Data source: provider surveys. Provider competence was transformed into logarithms. Response rates for each village clinic, township health centre, and county hospital were adjusted, and standard errors were clustered at the level of regions (townships).

## Discussion

This study modified the PHC quality framework to the context of rural China and evaluated the PHC quality for chronic diseases drawing on multiple data sources. We would like to highlight the following findings. With a focus on the "process" dimension, PHC quality for chronic diseases can be divided into three domains: quality of the PHC system, quality of clinical care, and quality of user experience. PHC quality in rural China reveals a nuanced situation marked by both strengths and shortcomings. Most quality sub-domains demonstrate favourable levels (scoring more than 0.7 out of 1); while continuity of care, treatment, provider competence, family-centeredness and shared decision-making have significant deficiencies. The differences among population subgroups in PHC quality, although statistically significant, are small concerning the gap between actual and optimal PHC quality. These results reflect the systematic nature of low-quality problems in low-resource areas. The primary challenge is to enhance the generally suboptimal PHC quality at the aggregate level. Our findings may also have implications for other low-resource settings with transitional health systems facing an increasing burden of chronic diseases.

The PHC system exhibits a satisfactory quality score concerning accessibility, comprehensiveness, and coordination, but a remarkably low score for continuity. We consider several possible explanations for this result. The achievements in accessibility reflected China’s efforts in the past two decades. Providing its citizens with equitable and affordable access to basic health care and financial protection has been set as a priority in national policies [40]. China has built a national social health insurance scheme covering nearly all populations, and catastrophic medical insurance and medical aid to strengthen financial protection for low-resource populations since 2009 [41]. Lacking a qualified health workforce in rural areas is a major challenge for improving accessibility, especially in low-resource western and central areas of China. To address this problem, China launched a national compulsory services program to train and provide qualified general practitioners with a five-year medical training program in these areas in 2010. The program has been implemented for more than 13 years and provides more than 5000 general practitioners each year [12]. China has also invested great efforts in improving comprehensiveness, notably the launch of a comprehensive national public health services program [27]. This program included the establishment of health records for all, health education, elderly care, care for major chronic diseases (including hypertension and type-2 diabetes) and infectious diseases, vaccinations, hygiene monitoring and more [27]. For patients with hypertension or type-2 diabetes, the service package includes screening, monitoring, routine follow-up and customized interventions [42]. PHC facilities provide these basic public health services to residents free of charge. Stable funding based on capitation from central government and local governments ensures the financing of the program. The scope of services provided by PHC has greatly expanded due to this program. A national evaluation study of this program showed that residents were widely satisfied with the service of PHC providers [42]. The achievements in coordination can be attributed to China’s massive investments in building a PHC information system. Health information systems can improve the quality of care by improving coordination as a direct mechanism [43]. China’s PHC facilities used to solely rely on paper-based medical records, and now nearly half of the rural facilities are using electronic information systems [42]. There are problems applying information technology in rural areas, for example, low willingness to adopt new information technology [44] and poor record-keeping behaviours [45].

The deficiency in continuity suggests a high fragmentation exists in the chronic care continuum. Two factors contribute to this problem. First, China’s current health system is criticized for being hospital-centric–the share of outpatient services in PHC facilities decreased from 62% in 2010 to 50% in 2021. Medical resources are concentrated in hospitals, leaving PHC facilities weak. Maintaining high continuity of care in hospital settings is challenging, especially for providers with heavy workloads to remember and build relationships with patients from various communities. Second, patients do not trust PHC facilities and turn to crowded hospitals for first-contact care [40]. Within the currently fragmented hospital-centric health system, it is difficult to build care continuity for chronic disease management and control. China has committed to enhancing PHC. Reforms including family physician care models and building an integrated delivery system were launched in recent years, but their impact on PHC quality was underexamined [26]. Enhancing continuity requires efforts targeting those two factors–decentralization of resources to PHC facilities and management of patient expectations.

We found that large gaps remained in treatment quality and provider competence, suggesting PHC providers are not prepared for the growing number of chronic patients and complex healthcare needs. Our findings are in line with pre-existing evidence [40, 46, 47]. UHC progress evaluation showed large gaps in non-communicable disease control and management in China as well as in many other low- and middle-income countries [40]. The treatment of chronic diseases is poor in rural China: a national survey estimated that in 2018 only 28.8% of rural patients with diabetes were treated (vs 36.2% in urban); only 44.1% of patients being treated were controlled (vs 54.1% in urban) [48]. For patients with hypertension, another national survey showed that in 2017 only 28.2% (vs 33.4% in urban) were taking prescribed antihypertensive medications [46]. The PHC quality in urban and rural areas is both unsatisfactory, but the situation is worse in the latter. China’s rural PHC providers have low levels of education and qualifications: in 2021, only 31.3% of the doctors in township health centres have college degrees and 17.5% in village clinics [47]. Without sufficient knowledge of chronic disease, it is impossible to improve clinical quality. Hospitals, medical universities, or associations should provide more training programs for PHC providers. Pairing assistance between secondary hospitals and PHC institutions will also benefit PHC providers’ improvement [49].

We found large gaps in shared decision-making and family-centeredness. An understanding of the illness and active participation in monitoring and treatment are essential for effectively managing chronic conditions. China’s PHC is largely based on ‘doctors’ orders–paternalistic advice with limited information sharing. As detailed in the WHO Innovative Care for Chronic Conditions model, a family-centred approach is important for the long-term management of chronic diseases because changing lifestyles is often difficult and needs family support [50]. Involving family members helps PHC doctors to better understand patients’ overall situation and to target social, emotional, and environmental determinants. However, rural PHC doctors lack awareness of family-centeredness and effective interventions in a family-centered way. China’s health care system should emphasize the role of family in caring for chronic diseases, especially changing lifestyles. Shared decision-making and family-centeredness can be taught through training programs.

Our findings that the PHC quality has significant but small variations in different sub-populations are in line with many published studies. PHC quality varies among patient characteristics and provider characteristics, but till now studies have not identified equity of quality as a primary problem. A study comparing the quality of care among sociodemographic groups in the US found that women had a 57% quality-of-care score, significantly higher than 52.3% of men (P<0.001)–but the difference is small compared to the general low-quality score [51]. Another study in India found that poor and rich people living in the same village received similar quality of care [13]. Studies have explored gender differences in the quality of care for decades but reached mixed conclusions [52].

We used multiple methods to assess quality: chart abstraction, patient survey, and vignette through provider survey. Results measured by chart abstraction or vignette may underestimate quality, as a result of incomplete documentation, especially for paper-based documentation, and underreporting information. The materials used in our study were primarily from the electronic health information system, which could minimize recording bias or inaccuracy. Chart abstraction has advantages over other methods of assessing quality–lower cost, more convenient access, and a large sample size. Vignette has proved both efficacy and efficiency in measuring quality. A study comparing different methods measuring the quality of care estimated that chart abstraction underreported quality 10.6 percent lower than standardized patients, and 5.4 percent lower than vignette [53]. The low quality of clinical care and provider competence can be partially explained by measurement methods, but the shortfalls are too widespread to be fully attributed to methods. Covering a wide range of sub-domains in PHC quality may compromise reliability to some extent. While Cronbach’s α values below 0.6 are acceptable for exploratory research [54, 55], future studies could consider balancing a higher reliability with a broader range of sub-domains.

This study is subject to several limitations. The PHC quality scores are primarily based on surveys, which may be subject to bias due to systematic variations in people’s expectations. As a result, even though the quality score is objectively the same, it may be assessed higher by people with lower expectations. Future research could try to measure and control people’s expectations of PHC quality. We mainly used hypertension and diabetes as tracer diseases. Excluding other major chronic diseases in China (i.e. cancer, and respiratory diseases) could lead to some loss of information. Future research could include a broader range of chronic diseases to better evaluate PHC quality. Furthermore, the findings of this study, conducted in three under-resourced counties, despite their implications to other low-resource settings, may not be generalizable to all of China. In addition, the data obtained from medical records may differently underrepresent actual quality due to potential incomplete documentation by providers. Measuring PHC quality from external surveys is less sustainable than using routine data such as medical records or other registries. Future studies could explore further measuring PHC quality using these data.

## Conclusion

We modified the PHC quality framework for chronic diseases and used it for assessing PHC quality in low-resource settings. PHC quality can be measured from three domains: PHC system, clinical quality and user experiences. The quality of chronic diseases in rural China has shown strengths and limitations. Significant deficiencies were identified in the PHC system (continuity of care), clinical care (treatment and provider competence) and use experience (family-centeredness and shared decision-making). Future efforts to improve PHC quality should be directed at these areas.

## Supporting information

S1 Appendix (DOCX)

S1 Checklist STROBE statement—checklist of items that should be included in reports of *cross-sectional studies*. (DOCX)
