# Supplementary material for: Quality of primary health care for chronic diseases in low-resource settings: Evidence from a comprehensive study in rural China
Source: PLoS One. 2024 Jul 25;19(7):e0304294. doi: 10.1371/journal.pone.0304294 (PMC11271947; doi:10.1371/journal.pone.0304294)
Supplement: S1 Appendix — (DOCX) [file pone.0304294.s001.docx]

**Appendix**

# **Table A1** Comparison of conceptual framework of PHC quality for chronic diseases in rural China with HQSS and PHCPI

| **HQSS** | **PHCPI*** | **Our study** |
| --- | --- | --- |
| **Competent systems**   - Safety - Prevention and detection - Continuity and integration - Population-health management - Timely action   **Evidence-based care**   - Systematic assessment - Correct diagnosis - Appropriate treatment - Counselling   **Positive user experience**   - Patient focus - Choice of provider - Short wait times - Patient voice and values - Affordability - Ease of use - Respect - Dignity, privacy, nondiscrimination - Autonomy - Confidentiality - Clear communication | **Access**   - Financial - geographical - Timeliness   **High-quality PHC**   - First-contact accessibility - Continuity - Comprehensiveness - Coordination - Person-centeredness   **Availability of effective PHC services**   - Provider availability - Provider motivation - Patient-provider respect and trust - Safety - Provider competence   **Population health management**   - Local priority setting - Community engagement - Empanelment - Proactive population outreach   **Facility organization and management**   - Team-based care organization - Facility management capability and leadership - Information systems - Performance measurement and management | **Competent PHC system**   - Accessibility - Comprehensiveness - Continuity - Coordination   **Effective clinical care**   - Assessment - Diagnosis - Treatment - Management - Provider competence   **Positive user experience**   - Information sharing - Shared decision-making - Respect for patients’ preferences - Family-centeredness |

**We focused on the service delivery section of PHCPI that corresponds to PHC quality in our study. HQSS-* *High-Quality Health System Framework*^1^*; PHCPI-* *Primary Health Care Performance Initiative Framework*^2^*.*

# **Table A2** Quality of primary healthcare component indicators selected from the comprehensive survey of China-Gates Primary Health Care Project

| **Sub-domains** | **Indicators** | | **Data sources** |
| --- | --- | --- | --- |
| **PHC system** |  | |  |
| *Accessibility* |  | |  |
| - Geographical access | - How far is the nearest health facility from your home? - How many minutes does it take, at the fastest, from your home to the nearest health facility? ("Fastest" refers to using easily accessible transportation, not limited to walking) | | Patient survey |
| - Financial access | - Do you have medical insurance? - What type of medical insurance do you have? (1) Urban Employee Basic Medical Insurance (2) Urban Resident Basic Medical Insurance (3) New Rural Cooperative Medical Care (4) Urban and Rural Resident Medical Insurance (5) Commercial Medical Insurance (6) Other Medical Insurance (7) No Medical Insurance - For patients seeking outpatient care or hospitalization at county-level hospitals and below, what is the out-of-pocket percentage? - For this medical visit, how much did the doctor or hospital charge you in total (i.e., total outpatient bill)? How much of it did you pay out of your own pocket? | | Patient survey |
| - Availiability | - During non-office hours at the hospital, such as evenings or weekends, can you inquire about health issues through phone, WeChat, text messages, etc.? - How long was the waiting time for your recent medical appointment? | | Patient survey |
| *Comprehensiveness* | | | |
| - Comprehensiveness | *Assistance in disease self-management:*   - In the past year, have you participated in any diabetes/hypertension self-management group activities?   *Health promotion and health education:*   - In the past year, has the doctor prescribed dietary interventions for you (such as dietary guidance like salt and oil restrictions)? - In the past year, has the doctor prescribed exercise interventions for you (such as exercise recommendations like daily step goals)?   *Guidance on drug usage:*   - In the past year, has the doctor verified the medications you are taking and discussed how to take them with you? | | Patient survey |
| *Conituity* |  | |  |
| - Relational contiuity | - Do you believe that the doctor most familiar to you understands your main health issues? - Do you believe that the doctor most familiar to you has a comprehensive understanding of your life background, habits, etc.? - Do you receive services from the same doctor every time you visit the township health center? | | Patient survey |
| - Informational continuity | - Do the doctors at the county hospital understand your health records (or follow-up information for hypertension/diabetes)? - Typically, when you visit the township health center, do the doctors there have knowledge of your treatment history at county hospitals or other hospitals? - Typically, when you visit the county hospital, do the doctors there have knowledge of your treatment history at township health centers or village clinics? | | Patient survey |
| - Management continuity | - When you were recently referred to the county hospital, did the doctor at the township health clinic assist you in making an appointment for registration? | | Patient survey |
| *Coordination* |  | |  |
| - Informational coodination | - Have you established a health record at township health center/community health center? | | Patient survey |
| - Service coordination | - Have you contracted for family doctor services? - When you were referred to the county hospital last time, did the doctor at the township health center discuss with you and recommend relevant departments or doctors? | | Patient survey |
|  |  | |  |
| **Clinical care** |  | |  |
| *Assessment* | // Diabetes   - Record smoking habits - Record alcohol drinking habits - Record family History - Measure height/BMI/weight - Measure waist circumference | // Hypertension   - Record smoking habits - Record alcohol drinking habits - Record family History - Measure height/BMI/weight - Record the highest blood pressure in the past - Record stratification of cardiovascular risk factors in hypertension | Chart abstraction |
| *Diagnosis* | //Diabetes   - Record the highest value of fasting blood glucose - Record the highest value of blood glucose 2 hours after meals - Perform glycated hemoglobin (HbA1c) test - Perform oral glucose tolerance test (OGTT) - Perform liver and kidney function tests - Perform blood lipid test - Perform oophthalmoscopic exam | //Hypertension   - Perform urinalysis - Perform electrocardiogram (ECG or EKG) - Perform blood glucose measurement - Perform blood blood pressure measurement - Perform blood pressure level classification - Perform blood homocysteine - Perform echocardiogram (ultrasound examination of the heart) | Chart abstraction |
| *Treatment* | //Diabetes   - Prescription: Biguanides, Sulfonylureas, Glinides, Alpha-glucosidase inhibitors, Insulin, Thiazolidinediones, DPP-4 inhibitors, SGLT2 inhibitors, GLP-1 receptor agonists. - Appropriate prescription - Adjust presciption - Appropriate indications for inpatient services - Referal | //Hypertension   - Presciption: diuretics, β-Blockers, calcium channel Blockers (CCB), Angiotensin-Converting Enzyme Inhibitors (ACEI), Angiotensin II Receptor Blockers (ARB) - Appropriate prescription - Adjust presciption - Appropriate indications for inpatient services - Referal | Chart abstraction |
| *Disease management* | //Diabetes   - Follow-up visits - In the last three months, how many times have you received follow-up guidance for diabetes? (1) zero (2) more than once - What services did you receive in your most recent diabetes follow-up? (1) Fasting blood glucose measurement (2) Lifestyle guidance (3) Inquiry about the medical condition (4) Inquiry about medication use | //Hypertension   - Follow-up visits - In the last three months, how many times have you received follow-up guidance for hypertension? (1) zero (2) more than once - What services did you receive in your most recent hypertension follow-up? (1) Blood pressure measurement (2) Lifestyle guidance (3) Inquiry about the medical condition (4) Inquiry about medication use | Patient survey, national register data of basic public health services, |
| *Provider competence* | | | |
| - Medical knowledge | //Diabetes   - Knowledge of diagnosis   - Correct diagnosis of diabetes   - Knowledge of assessment and treatment   - Recommend to control weight  - Recommend to have a balanced diet  - Recommend to engage in appropriate exercise  - Recommend to quit smoking/reduce alcohol consumption  - Recommend to use oral medications  - Recommend to use metformin for treatment  - Correct recommended dosage of metformin  - Recommend self-blood glucose monitoring  - Recommend to initiate insulin if blood glucose control is not achieved  - Recommend referral/additional diagnostic tests   - Knowledge of disease management   - Recommend to establish health records/included chronic disease follow-up management  - Recommend regular follow-up management  - Ask complications/low blood sugar reactions  - Recommend regular blood glucose tests  - Recommend regular physical examinations | //Hypertension   - Knowledge of diagnosis   - Correct diagnosis of levles of hypertension   - Knowledge of assessment and treatment   - Recommend to control weight  - Recommend to have a balanced diet  - Recommend to engage in appropriate exercise  - Recommend to quit smoking/reduce alcohol consumption  - Recommend to use medications  - Recommend to use Angiotensin-converting enzyme inhibitors (ACEI) or Angiotensin II receptor blockers (ARBs)  - Correct recommended dosage of ACEI/ARB  - Recommend to use diuretics  - Correct recommended dosage of diuretics  - Recommend to reduce mental stress and maintain psychological balance  - Recommend referral/additional diagnostic tests   - Knowledge of disease management   - Recommend to establish health records/included chronic disease follow-up management  - Recommend regular follow-up management  - Ask complications  - Recommend regular blood pressure measurement  - Recommend regular physical examinations | Provider survey |
|  |  | |  |
| **User experience** |  | |  |
| *Information sharing* | - How do you feel about the attentiveness of the doctor in listening to your description of the illness during this visit? - How clear was the explanation of the treatment plan by the doctor to you? - What is your opinion on the carelfuness with which the doctor listened to your description of the illness during this hospitalization? - How clear was the explanation of the treatment plan by the doctor staff to you? | | Patient survey，provider survey |
| *Shared decision-making* | - Do the doctors at the township health center seek your opinion when formulating a treatment plan for you? - Do the doctors at the county hospital seek your opinion when formulating a treatment plan for you? | | Patient survey，provider survey |
| *Respect for patients’ preferences* | - Do you feel that your privacy was respected during the course of your medical treatment? - Did the medical staff give you sufficient time to ask questions about your condition or treatment during the outpatient visit? - Do you believe that your privacy was respected during your hospitalization? - Did the doctor give you sufficient time to ask questions about your condition or treatment during your hospital stay? | | Patient survey，provider survey |
| *Family-centeredness* | - Did the doctor at the township health center ask about whether your family members have a family medical history or other health issues? - Did the doctor at county hospital ask about whether your family members have a family medical history or other health issues? - During the most recent follow-up, did the doctor provide health guidance for other members of your family? | | Patient survey，provider survey |

# **Table A3** The social economic background of three counties

| Social economic background of three counties | Hubei | Henan | Shanxi | China |
| --- | --- | --- | --- | --- |
| Per capita GDP (CNY) | 29693 | 60442 | 63409 | 85698 |
| Population (in ten thousand people) | 38.37 | 56.60 | 12.76 | 141175 |
| Per capita disposable annual income of urban residents (CNY) | 33928 | 35868 | 39031 | 49283 |
| Per capita disposable annual income of rural residents (CNY) | 14313 | 15452 | 13838 | 20133 |
| Hospital beds per thousand population | 7.72 | 7.70 | 12.28 | 6.92 |
| Number of doctors per thousand population | 2.88 | 2.38 | 2.72 | 2.90 |

*Notes: (1) Source：Statistical bulletin of three counties 2022, National Bureau of Statistics of China; (2) GDP per capita, disposable income (ten thousand CNY in 2020)*

|  | PHC system | | | | Clinical care | | | | | User experience | | | |
| --- | --- | --- | --- | --- | --- | --- | --- | --- | --- | --- | --- | --- | --- |
|  | Accessibility | Comprehensiveness | Continuity | Coordination | Assessment | Diagnosis | Treatment | Disease management | Medical knowledge* | Shared decision-making | Family-centeredness | Information sharing | Respect for patients' preferences |
| Gender^#^ | 0.787 | 0.033 | 0.099 | 0.007 | 0.292 | 0.483 | 0.754 | 0.281 | 0.098 | 0.170 | 0.113 | 0.466 | 0.336 |
| Age | <0.001 | 0.087 | 0.076 | 0.001 | 0.016 | 0.026 | 0.408 | 0.063 | 0.519 | 0.959 | <0.001 | <0.001 | <0.001 |
| Marriage^#^ | 0.023 | 0.747 | 0.913 | 0.429 | 0.754 | 0.149 | 0.319 | 0.207 | 0.123 | 0.893 | <0.001 | 0.624 | 0.393 |
| Education | <0.001 | <0.001 | 0.080 | <0.001 | 0.333 | 0.004 | 0.480 | 0.340 | 1.000 | 0.915 | 0.195 | 0.091 | <0.001 |
| Income group | <0.001 | 0.003 | 0.123 | 0.001 | 0.277 | 0.045 | 0.670 | 0.002 | 0.122 | 0.522 | 0.002 | <0.001 | <0.001 |
| Medical insurance | <0.001 | <0.001 | <0.001 | <0.001 | 0.001 | 0.070 | 0.466 | <0.001 | NA | 0.418 | <0.001 | <0.001 | <0.001 |

# **Figure A1** P-values of statistical tests in PHC quality score distribution by social-economic characteristics

***Notes:*** *^#^ T-tests were used to examine the difference of the quality score for gender and marriage groups; ANOVA was used for other socio-economic variables. P<0.05 was considered as statistically significant.*

# **Table A4** Evidence of reliability and validity of PHC quality domains and sub-domains

| **Domain and sub-domain** | **Reliability or validity tools** |
| --- | --- |
| **PHC system** | Scale reliability coefficient (Cronbach's α) |
| Accessibility | 0.551 |
| Comprehensiveness | 0.616 |
| Continuity | 0.754 |
| Coordination | 0.767 |
| Total | 0.583 |
| **Clinical Care** | **Clinical guidelines and expert opinions** |
| Assessment | Chinese Guidelines for the Prevention and Treatment of Hypertension (Revised Edition 2018); Chinese Guidelines for the Prevention and Treatment of Type 2 Diabetes (2020 Edition); Chinese Guidelines for the Diagnosis and Treatment of Elderly Diabetes (2021 Edition);  Expert opinions of GPs and internists |
| Diagnosis | Chinese Guidelines for the Prevention and Treatment of Hypertension (Revised Edition 2018); Chinese Guidelines for the Prevention and Treatment of Type 2 Diabetes (2020 Edition); Chinese Guidelines for the Diagnosis and Treatment of Elderly Diabetes (2021 Edition);  Expert opinions of GPs and internists |
| Treatment | Guidelines for Rational Use of Antihypertensive Medications (2nd Edition);  Chinese Guidelines for the Prevention and Treatment of Hypertension (Revised Edition 2018) Chinese Guidelines for the Prevention and Treatment of Type 2 Diabetes (2020 Edition) Guidelines for Rational Use of Medications for Type II Diabetes;  Chinese Guidelines for the Diagnosis and Treatment of Elderly Diabetes (2021 Edition);  Expert opinions of GPs and internists |
| Disease management | Chinese Guidelines for Primary Care Management of Hypertension (Revised Edition 2014); National Basic Public Health Service Standards (Third Edition);  Expert opinions of GPs and internists |
| Provider competence | 0.711 (Cronbach's α)  Expert opinions of GPs and internists |
| **User experience** | Scale reliability coefficient (Cronbach's α) |
| Shared decision-making | 0.720 |
| Family-centeredness | 0.548 |
| Information sharing | 0.842 |
| Respect for patients' preferences | 0.831 |
| Total | 0.818 |

# **Table A5** Quality Evaluation Checklist for Type II Diabetes Assessment, Diagnosis, and Treatment

______ County (County Hospital □ Township Health Center □)

Medical Record Number___________

| Name： | | | Gender: | | | | Age： | | | ID Card Number： | | | |
| --- | --- | --- | --- | --- | --- | --- | --- | --- | --- | --- | --- | --- | --- |
| Diabetes first diagnosed: _______ year. Admission time: _______, _______, _______. | | | | | | | | | | | | | |
| Measuring Blood Glucose | | | Not Done □  Done□ | | | | The highest fasting blood glucose level (mmol/L): | | | | | | |
|  |  |  |  |  |  |  | The highest 2-hour postprandial blood glucose level (mmol/L): | | | | | | |
| Unhealthy Lifestyle Habits | | | Smoking: Yes □ No □ Not Filled □ | | | | | Alcohol Consumption: Yes □ No □ Not Filled □ | | | | | |
| Family Medical History | | | Yes □ No □ Not Filled □ | | | | | | | | | | |
| Height (cm): | Weight (Kg): | | | | | | AC（cm） | | | | | BMI（Kg/m2） | |
| Hemoglobin A1c (HbA1c) Test | | Not Done □ Done □ | | | | | Most Recent Result: | | | | | | |
| Oral Glucose Tolerance Test (OGTT) | | | | Not Done □ | | | | | | Done □ | | | |
| Urinalysis | | | WBC: Positive □ Negative □ Not Done □ | | | | | | | PRO: Positive □ Negative □ Not Done □ | | | |
| Liver Function and Kidney Function Tests | | | Not Done □ | | | | | | | Done □ | | | |
| Lipid Monitoring | | | Not Done □ | | | | | | | Done □ | | | |
| ECG | | | Not Done □  Done □ | | | Conclusion: ______________________________ | | | | | | | |
| Fundoscopy | | | Not Done □  Done □ | | | Conclusion: ______________________________ | | | | | | | |
| Prescription | | | Biguanides□ | | Sulfonylureas□ | | | | Glinides□ | α- Glucosidase Inhibitors□ | | | Insulin □ |
|  |  |  | Thiazolidinediones□ | | DPP-4 Inhibitors□ | | | SGLT2 Inhibitors□ | | | GLP-1 Receptor Agonists□ | | |
| Appropriateness of prescription | | | Reasonable □ | | | | | | | Unreasonable □ | | | |
| Adjustment of prescription | | | Yes □ | | | | | | | No □ | | | |
| Lifestyle Guidance | | | Yes □ | | | | | | | No □ | | | |
| Complications | | | No□ Infectious Disease□ | | | | Cardiovascular Disease□ | | | Diabetic Nephropathy□ | | | Diabetic Foot□ |
|  |  |  | Diabetic Retinopathy□ | | | | Acute Severe Metabolic Disorder□ | | | | Neurological Complications□ | | |
| Indication for admission | | | | Yes □ | | | | | | No □ | | | |
| Blood glucose control status | | | ______________________________________ | | | | | | | | | | |
| Referral required | | | Yes□ No□ | | | Destination：______________________________ | | | | | | | |
| Note: The target for fasting blood glucose control is 4.4-7.0 mmol/L, and for non-fasting blood glucose is ≤10.0 mmol/L. Glycated hemoglobin should be <7.0 mmol/L. | | | | | | | | | | | | | |

# **Table A6** Quality Evaluation Checklist for Hypertension Assessment, Diagnosis, and Treatment

______ County (County Hospital □ Township Health Center □)

Medical Record Number___________

| Name： | Gender: | Age： | | | | ID Card Number： | | | | | | | |
| --- | --- | --- | --- | --- | --- | --- | --- | --- | --- | --- | --- | --- | --- |
| Hypertension first diagnosed：_____Year | | The highest blood pressure ever (mmHg): | | | | | | | | | | | |
| Date of admission: Year ______ Month ______ Day _______ | | | | | | | | | | | | | |
| Blood pressure measurement | Not done□ Done□ | | Blood pressure of left upper limb(mmHg)： | | | | | | | Blood pressure of right upper limb (mmHg): | | | |
| Blood pressure level | Level 1□ | | | Level 2□ | | | | | | | Level 3□ | | |
| Cardiovascular risk stratification | Low risk□ | | | | | | | Moderate risk □ | | | | | |
|  | High risk□ | | | | | | | Very high risk□ | | | | | |
| Unhealthy lifestyle | Smoking: Yes□ No□ Not filled□ | | | | | | | Drinking: Yes□ No□ Not filled□ | | | | | |
| Family medical history | Yes□ No□ Not filled□ | | | Height (cm): | | | | Weight (Kg): | | | | | BMI: |
| Serum homocysteine | Not Done□  Done□ | | | Conclusion: | | | | | | | | | |
| Biochemical indicators | Not Done□  Done□ | | | Urine occult blood  Negative□ Positive□ | | | | | | | | PRO  Negative□ Positive□ | |
| ECG | Not Done□  Done□ | | | Conclusion: | | | | | | | | | |
| Echocardiogram | Not Done□  Done□ | | | Conclusion: | | | | | | | | | |
| Prescription | Diuretics□ | | | | Β-blockers□ | | | | | | CCB□ | | |
|  | ACEI □ | | | | | | | ARB □ | | | | | |
| Reasonableness of Prescription | Reasonable □ | | | | | | | | Unreasonable □ | | | | |
| Adjustment of prescription | Yes □ | | | | | | | | No □ | | | | |
| Lifestyle Guidance | Yes □ | | | | | | | | No □ | | | | |
| Complications | None□ Cerebrovascular disease□ | | | | | | | Heart failure and coronary heart disease□ | | | | | |
|  | Peripheral vascular stenosis□  Chronic renal failure□ | | | | | | | Aortic dissection□ | | | | | |
| Indication for admission | Yes □ | | | | | | | No □ | | | | | |
| Treatment outcome: |  | | | | | | | | | | | | |
| Referral | Yes□ No□ | | | | | | Destination__________________________ | | | | | | |
| Note: The target for blood pressure control is <140/90 mmHg. For patients comorbid with diabetes, CKD, heart failure, or stable coronary heart disease, the target is <130/80 mmHg. For elderly patients with isolated systolic hypertension, systolic BP should be <150 mmHg, and if tolerated, <140 mmHg. | | | | | | | | | | | | | |

**Table A7**

**Patient survey questionnaire**

**County code □□□□□□ Township code □**

**Village code □ Person code □□□□**

**Basic information**

1.name：__________________ 2.ID：_____________________________________ 3.phone：_______________________

4.address：__________________county__________________township__________________village

5. disease： hypertension□ diabetes□ comorbid with hypertension and diabetes□

Name of interviewer： _________________

**Table 1. Family information**

| No. | Questions | Answer |
| --- | --- | --- |
|  | How many people usually live together in your household? (i.e., permanent residents) |  |
|  | How far is the nearest medical facility from your home? (1) Less than 1 kilometer (2) 1-5 kilometers (3) 5 kilometers or more |  |
|  | How long does it take to get from your home to the nearest medical institution? ("Fastest" means using the most readily available means of transportation rather than solely walking) |  |
|  | If you have a minor illness, how do you usually deal with it? (1) See a doctor (2) Buy some medicine yourself (skip to question 6) (3) Ignore it and wait for it to get better on its own (skip to question 6) (4) Other (skip to question 6) |  |
|  | Where do you usually go first if you have a minor illness? (1) Village clinic (2) Private clinic (3) Township health center (4) County public hospital (5) County private hospital (6) County-level hospital outside the county (7) City-level or provincial hospital outside the county (8) Other |  |
|  | Is your household registered as a poverty-stricken household? (1) Yes (2) No (3) Don't know |  |
|  | Is your household classified as a low-income household? (1) Yes (2) No (3) Don't know |  |
|  | If your household is classified as a poverty-stricken or low-income household, what do you think is the main reason for poverty in your household? (1) Lack of labor force (2) Poor natural conditions or disasters (3) Illness or injury affecting labor capacity (4) Spending a lot of money on medical treatment (5) Unemployment (6) Human factors (7) Other |  |
|  | Has your household already been lifted out of poverty? (1) Yes (2) No (3) Don't know |  |
|  | What was the total income of your household in the previous year (2021)? (Including income from wages, business, property, and various forms of transfer income, net of personal income tax, social security expenses, support expenses, interest expenses, etc., excluding financial sales and borrowing income, as well as inheritance or one-time gift income) |  |
|  | What was the total household expenditure in the previous year (2021)? (Including expenditure on food, tobacco and alcohol, clothing, housing, household goods and services, transportation and communication, education, culture, entertainment, healthcare, and other items, excluding social security expenses, purchase of commercial insurance, expenditure on marriage, funeral, and other ceremonial expenses, as well as expenditure on purchasing or building houses and other non-consumption expenditures) |  |
|  | How much was the expenditure on food in your household in the previous year (2021)? (Including grains, cooking oil, vegetables, meat, fruits, milk powder, snacks, etc., excluding expenses on tobacco, alcohol, dining out, etc.) |  |
|  | How much did your household directly pay for medical services in the previous year (2021)? (Excluding expenses that have already been reimbursed or are expected to be reimbursed) |  |
|  | How much did your household spend on healthcare (including healthcare equipment, supplies, and services) in the previous year (2021)? (Including purchases of nutritional supplements, blood pressure and blood glucose monitors, massage therapy, family planning supplies, etc.) |  |

**Table 2. Personal information**

| No. | Questions | Answer |
| --- | --- | --- |
|  | In your household, are you:   1. The head of the household 2. The head's spouse 3. The head's child 4. The head's son-in-law or daughter-in-law 5. The head's parent 6. The head's parent-in-law 7. The head's grandparent 8. The head's grandchild 9. The head's sibling 10. Other |  |
|  | Gender:   1. Male 2. Female |  |
|  | Ethnicity: ⑴ Han (2) Other ethnicity (please specify): __________________ |  |
|  | Age：___________ |  |
|  | Education level: ⑴ Primary school or below ⑵ Junior high school ⑶ Senior high school ⑷ Vocational school or technical secondary school ⑸ College or above) |  |
|  | Marital status: ⑴ Unmarried ⑵ Married ⑶ Widowed ⑷ Divorced |  |
|  | What is your current occupation?  (1) Farming (2) Unemployed (3) Working as an employee (4) Self-employed (5) Professional technical personnel (including rural teachers, village doctors, etc.) (6) Grassroots workers (such as village cadres, etc.) (7) Retired (8) Other |  |
|  | What type of medical insurance do you have? (1) Urban employee basic medical insurance (2) Urban resident basic medical insurance (3) New rural cooperative medical scheme (4) Urban and rural resident medical insurance (5) Commercial medical insurance (6) Other medical insurance (7) No medical insurance |  |
|  | Are you currently receiving government medical assistance? ⑴ Yes ⑵ No (3) Not sure |  |
|  | Please rate your health status today on a scale from 0 to 100:  ├—┼—┼—┼—┼—┼—┼—┼—┼—┼—┤  0 10 20 30　40 50 60 70 80 90 100  Worst health condition Best health condition |  |
|  | Do you smoke? ⑴ Every day ⑵ Not every day ⑶ Used to smoke, but now quit (4) Never smoked |  |
|  | Have you consumed alcohol in the past month? (1) At least 3 times a week ⑵ Less than 3 times a week ⑶ Never drank |  |
|  | Have you established a health record at a township health center/community health institution? (1) Yes (2) No, but aware of this service (3) Not sure |  |
|  | Have you signed up for family doctor services? (1) Yes (2) No, but aware of this service (3) Not sure |  |
|  | Can you consult about health issues via phone, WeChat, text messages, etc., outside of normal hospital working hours (such as evenings, weekends)? (1) Yes (2) No |  |
|  | Do you have any other diagnosed chronic diseases besides hypertension or diabetes? (1) Yes ⑵ No |  |
|  | In the past year, have you participated in diabetes/hypertension self-management group activities? (1) Yes (2) No |  |
|  | In the past year, has a doctor prescribed dietary intervention (dietary guidance, such as salt and oil restriction) for you? (1) Yes (2) No |  |
|  | In the past year, has a doctor prescribed dietary intervention (dietary guidance, such as salt and oil restriction) for your family members? (1) Yes (2) No |  |
|  | Who prescribed the dietary intervention? (1) County hospital doctor (2) Township health center doctor (3) Township health center public health doctor (4) Village doctor (5) Other: ____________ |  |
|  | In the past year, has a doctor prescribed exercise intervention (exercise recommendations, such as daily steps) for you? (1) Yes (2) No |  |
|  | In the past year, has a doctor prescribed exercise intervention (exercise recommendations, such as daily steps) for your family members? (1) Yes (2) No |  |
|  | Who prescribed the exercise intervention?  (1) County hospital doctor (2) Township health center doctor (3) Township health center public health doctor (4) Village doctor (5) Other: ____________ |  |
|  | In the past year, has a doctor reviewed the medications you are taking and discussed how to take them? (1) Yes (2) No |  |
|  | Have you engaged in self-health management behaviors based on doctor's advice? (1) Regular exercise (2) Low oil, low salt, low-fat diet (3) Quit smoking, limit alcohol (4) Actively seek health information (5) Regular medical check-ups (6) None |  |
|  | Do doctors at the township health center consult you when devising treatment plans for you? (1) Never (2) Occasionally (3) Sometimes (4) Often (5) Always |  |
|  | Do doctors at the county hospital consult you when devising treatment plans for you? (1) Never (2) Occasionally (3) Sometimes (4) Often (5) Always |  |
|  | Do medical staff at the township health center inquire about your family members' family medical history or other health issues? (1) Never (2) Occasionally (3) Sometimes (4) Often (5) Always |  |
|  | Do medical staff at the county hospital inquire about your family members' family medical history or other health issues? (1) Never (2) Occasionally (3) Sometimes (4) Often (5) Always |  |
|  | Do you think the doctor or nurse who is most familiar with you understands your main health issues well? (1) Extremely well (2) Very well (3) Moderately well (4) Not very well (5) Not at all |  |
|  | Do you think the doctor who is most familiar with you understands your lifestyle background, habits, etc. comprehensively? (1) Extremely well (2) Very well (3) Moderately well (4) Not very well (5) Not at all |  |
|  | Do you prefer to be served by the same doctor every time you visit the township health center? (1) Yes (2) No (3) No preference |  |
|  | Do doctors at the county hospital understand the information in your health records (or hypertension/diabetes follow-up)? (1) Extremely well (2) Very well (3) Moderately well (4) Not very well (5) Not at all |  |
|  | Generally, when you visit the township health center, do doctors there know about your treatment at the county hospital? (1) Extremely well (2) Very well (3) Moderately well (4) Not very well (5) Not at all |  |
|  | Do you receive services from the same doctor every time you visit the township health center? (1) Never (2) Occasionally (3) Sometimes (4) Often (5) Always |  |
|  | Generally, when you visit the county hospital, do doctors there know about your treatment at the township health center? (1) Never (2) Occasionally (3) Sometimes (4) Often (5) Always |  |
| **Chronic diseases** | | |
| **For patients with hypertension, please answer questions 51-61.** | | |
|  | How often do you take antihypertensive medication? (1) As prescribed by the doctor every day ⑵ Occasionally or as needed ⑶ Never |  |
|  | Did you measure your blood pressure yesterday? ⑴ Did not measure ⑵ Measured (fill in blood pressure value): __________ |  |
|  | When was the last time you measured your blood pressure?  (1) Within a week ⑵ Within a month ⑶ Within three months ⑷ Within six months ⑸ More than six months ago |  |
|  | Is your current (most recent measurement) blood pressure normal? (1) Yes (2) No (3) Don't know |  |
|  | In the last three months, has the doctor provided follow-up guidance for your hypertension?  (1) Conducted follow-up and guidance (2) Did not conduct follow-up and guidance |  |
|  | How many times have you received follow-up guidance for hypertension in the last three months?  (1) Once (2) Twice (3) Three times or more (6) Not followed up |  |
|  | What were the main contents of the last hypertension follow-up service you received?  (1) Blood pressure measurement (2) Lifestyle guidance (3) Inquiry about disease status (4) Inquiry about medication usage |  |
|  | What form of hypertension follow-up service did you receive most recently? (Including active medical consultation, but excluding only buying medication after visiting a medical institution without receiving other services) (1) Contracted family doctor home visit follow-up (2) Other medical staff home visit follow-up (3) Medical institution visit or follow-up (4) Telephone follow-up (5) Online follow-up (mobile app, etc.) (6) Other |  |
|  | During the last follow-up, did the doctor provide health guidance for other members of your household? (1) Yes (2) No |  |
|  | How many times have you seen a doctor for hypertension in the last month? _________ |  |
|  | Do you have the following comorbidities of hypertension? (1) Cerebrovascular disease (2) Heart failure (3) Coronary heart disease (4) Renal failure (5) Aortic dissection (6) Other: _________ |  |
| **For patients with diabetes, please answer questions 62-74** | | |
|  | What type of diabetes do you have? (1) Type I diabetes (2) Type II diabetes (3) Don't know |  |
|  | How often do you take antidiabetic medication (or inject insulin)? (1) As prescribed by the doctor every day or every meal ⑵ Occasionally or as needed ⑶ Never |  |
|  | When was the last time you measured your blood sugar (including measurements by doctors/nurses and self-measurements)? (1) Within a week ⑵ Within a month ⑶ Within three months ⑷ Within six months ⑸ More than six months ago |  |
|  | Did you measure your blood sugar yesterday? (1) Did not measure ⑵ Measured (fill in blood sugar value________, fasting□ non-fasting□) |  |
|  | Is your current (most recent measurement) fasting blood sugar normal? (1) Yes (2) No (3) Don't know |  |
|  | In the last three months, has the doctor provided follow-up guidance for your diabetes? (1) Conducted follow-up and guidance (2) Did not conduct follow-up and guidance |  |
|  | How many times have you received follow-up guidance for diabetes in the last three months? (1) Once (2) Twice (3) Three times or more (4) Not followed up |  |
|  | What were the main contents of the last diabetes follow-up service you received? (1) Fasting blood sugar measurement (2) Lifestyle guidance (3) Inquiry about disease status (4) Understanding medication usage |  |
|  | What form of diabetes follow-up service did you receive most recently? (Including active medical consultation, but excluding only buying medication after visiting a medical institution without receiving other services) (1) Contracted family doctor home visit follow-up (2) Other medical staff home visit follow-up (3) Medical institution visit or follow-up (4) Telephone follow-up (5) Online follow-up (mobile app, etc.) (6) Other |  |
|  | During the last follow-up, did the doctor provide health guidance for other members of your household? (1) Yes (2) No |  |
|  | How many times have you seen a doctor for diabetes in the last month? _______________ |  |
|  | Do you have the following complications of diabetes? (1) Diabetic nephropathy (2) Diabetic neuropathy (3) Blindness (4) Diabetic foot (5) Coronary heart disease (6) Stroke (7) Amputation (8) Infectious diseases (5) Other: _________ |  |
| **The following questions are about your most recent visit:** | | |
|  | Where did you first see the doctor (i.e., the initial medical institution)? ⑴ Village health center ⑵ Private clinic ⑶ Clinic ⑷ Public hospital in the county ⑸ Private hospital in the county ⑹ County-level hospital outside the county ⑺ City or provincial hospital outside the county ⑻ Other |  |
|  | How long did you wait for medical treatment this time? (1) No need to wait (2) Less than 15 minutes (3) 15-30 minutes (4) More than 30 minutes |  |
|  | If the doctor said they couldn't treat you, where did you go afterwards? (Patients who received treatment at the initial medical institution do not need to answer)  (1) Clinic (2) Public hospital in the county (3) Private hospital in the county (4) County-level hospital outside the county (5) City or provincial hospital outside the county (6) Other |  |
|  | How much did the doctor or hospital charge you for this visit (total outpatient fee)?______________ |  |
|  | Among them: how much did you pay yourself (excluding reimbursement and personal medical account expenditures)?__________ |  |
|  | How much did you and the person accompanying you spend for this visit (e.g., transportation, food, accommodation)?______________ |  |
|  | How do you feel about the cost of this visit? (1) Very expensive (2) Expensive (3) Average (4) Not expensive (5) Not expensive at all |  |
|  | In the past month, have you needed to see a doctor for hypertension/diabetes but didn’t go? ⑴ Yes (skip to question 82) ⑵ No (skip to question 83) |  |
|  | The main reason for not seeing a doctor (choose one): (1) Inconvenient to seek medical treatment ⑵ No money for medical treatment ⑶ No time ⑷ Inconvenient transportation ⑸ Treatment seems ineffective ⑹ Other |  |
|  | How well do you think the medical staff listened to your description of your condition during this visit? (1) Very well (2) Well (3) Average (4) Not well (5) Not well at all |  |
|  | Do you feel your privacy was respected during the visit? (1) Very well (2) Well (3) Average (4) Not well (5) Not well at all |  |
|  | Did the medical staff give you enough time to ask questions about your condition or treatment? (1) Very well (2) Well (3) Average (4) Not well (5) Not well at all |  |
|  | How clear was the explanation of the treatment plan by the medical staff? (1) Very clear (2) Clear (3) Average (4) Not clear (5) Not clear at all |  |
|  | How satisfied are you with this visit overall? (1) Extremely satisfied (skip to question 89) ⑵ Very satisfied (skip to question 89) ⑶ Somewhat satisfied (skip to question 89) (4) Not very satisfied (skip to question 88) (5) Not satisfied at all (skip to question 88) |  |
|  | Among them, what are you most dissatisfied with (choose one): (1) Technical proficiency ⑵ Equipment conditions ⑶ Variety of medications ⑷ Service attitude ⑸ Medical expenses ⑹ Admission procedures ⑺ Waiting time ⑻ Environmental conditions ⑼ Provision of unnecessary services (including medication and tests) ⑽ Other |  |
| **The following questions are about your most recent referral:** | | |
|  | When you were last referred to the county hospital, did the doctor at the township health center discuss and recommend relevant departments or doctors with you? (1) Yes (2) No |  |
|  | When you were last referred to the county hospital, could the doctor at the township health center help you make an appointment for registration? (1) Yes (2) No |  |
|  | What do you think is the most important aspect of being referred to the county hospital? (choose one):  (1) Discussing and recommending relevant departments and doctors (2) Helping to make an appointment for registration (3) Stating the reason for the referral (4) Monitoring the treatment at the county hospital (5) Other _________ |  |

**Table 3. Inpatient visit**

| No. | Questions | Answer |
| --- | --- | --- |
|  | In the past 12 months, have you been hospitalized due to illness, injury, physical examination, childbirth, or other reasons? (1) Yes (2) No |  |
|  | In the past 12 months, has any doctor recommended hospitalization for you, but you did not go to the hospital?  (1) Yes (skip to question 94) (2) No (skip to question 95) |  |
|  | What was the main reason for your most recent need for hospitalization but not being hospitalized? (Choose one): (1) Hospitalization is troublesome (2) Lack of money for hospitalization (3) No time (4) Hospital too far away (5) Hospitalization won't help (6) Minor illness does not require hospitalization (7) Hospital has no available beds (8) Personal disregard (9) Other |  |
|  | Date of your most recent hospitalization: (Year) _________ |  |
|  | (Month) _________ |  |
|  | Where did you stay during this hospitalization? (1) Health center (2) County public hospital (3) County private hospital (4) Hospital outside the county level (5) Hospital outside the city or provincial level (6) Other |  |
|  | How many days did you stay in the hospital? _________ |  |
|  | Reason for discharge: (1) Recovered from illness, discharged by doctor's order (skip to question 101) (2) Illness not cured, doctor requested transfer to another hospital (skip to question 101) (3) Requested discharge by yourself (4) Other (skip to question 101) |  |
|  | If you requested discharge by yourself, what was the reason? (1) Illness remained uncured (2) Believed illness was already cured (3) Hospitalization cost too much (4) Poor hospital conditions (5) Poor hospital service attitude (6) Poor doctor's skills (7) Other |  |
|  | Total medical expenses for this hospitalization: _________ |  |
|  | Among them: How much did you bear personally? (excluding reimbursements and expenditures from personal medical accounts) _________ |  |
|  | The expenses for tests, surgeries, medications, consumables, etc., outside the hospital during your hospitalization this time amounted to _________ yuan. |  |
|  | How do you feel about the medical expenses for this hospitalization? (1) Very expensive (2) Expensive (3) Average (4) Not expensive (5) Not expensive at all |  |
|  | How much did you and the person accompanying you spend during this hospitalization (e.g., transportation, accommodation, meals, care)? _________ |  |
|  | During your hospitalization, how many days did the family member accompanying you miss work? _________ |  |
|  | How do you rate the attentiveness of medical staff to your description of the illness during your hospitalization? (1) Very good (2) Good (3) Average (4) Not good (5) Very poor |  |
|  | Do you feel that your privacy was respected during your hospitalization?  (1) Very good (2) Good (3) Average (4) Not good (5) Very poor |  |
|  | Did the medical staff give you adequate time to ask questions about your condition or treatment during your hospitalization? (1) Very good (2) Good (3) Average (4) Not good (5) Very poor |  |
|  | How clear were the explanations provided by the medical staff regarding the treatment plan during your hospitalization? (1) Very clear (2) Clear (3) Average (4) Not clear (5) Very unclear |  |
|  | How satisfied are you overall with this hospitalization? (1) Extremely satisfied (2) Very satisfied (3) Somewhat satisfied (4) Not very satisfied (5) Not satisfied at all |  |
|  | What are you least satisfied with? (Choose one) (1) Technical skills (2) Equipment conditions (3) Variety of medications (4) Service attitude (5) Medical expenses (6) Procedures for seeing a doctor (7) Waiting time (8) Environmental conditions (9) Provision of unnecessary services (including medications and tests) (10) Other |  |

**Doctor survey questionnaire**

**County code □□□□□□ Township code □**

**Village code □ Person code □□□□**

1. Person type：village doctor□ township health center - doctor □ township health center – public health doctor □ county hospital-doctor□
2. phone________________
3. institution：______________________________

4.workplace：__________province__________city__________county__________township

Name of interviewer： _________________

| No | Questions | Answers |
| --- | --- | --- |
| Basic information | |  |
|  | Gender: 1. Male 2. Female |  |
|  | Ethnicity: ⑴ Han (2) Other ethnicity (please specify): __________________ |  |
|  | Age：___________ |  |
|  | Education level: ⑴ Primary school or below ⑵ Junior high school ⑶ Senior high school ⑷ Vocational school or technical secondary school ⑸ College or above) |  |
|  | Marital status: ⑴ Unmarried ⑵ Married ⑶ Widowed ⑷ Divorced |  |
|  | Length of work experience: ________ years; Length of work experience in current position: ________ years |  |
|  | Do you currently have a permanent position? (1) Yes (2) No |  |
|  | Have you passed the physician qualification examination? (1) Yes (2) No (skip to 10) |  |
|  | Your physician qualification is: (1) Clinical Physician (2) Clinical Assistant Physician (3) Rural General Practice Assistant Physician (4) Dental Physician (5) Dental Assistant Physician (6) Public Health Physician (7) Public Health Assistant Physician (8) Other |  |
|  | Your professional title is: (1) No professional title (2) Junior (3) Intermediate (4) Associate Chief (5) Chief |  |
|  | Your administrative position in this unit is: (1) No administrative position (2) Dean/Secretary (3) Vice Dean/Vice Secretary (4) Department Director (5) Deputy Department Director (6) Other |  |
|  | In routine outpatient services, how many patients did you treat per day in the past week? ________ people/day |  |
|  | Among them, on average, how long is your treatment time per patient? _____ minutes |  |
|  | In routine inpatient services, on average, how many hospitalized patients do you manage per month in the past year? ________ patients/month |  |
|  | Your total monthly income is: ________ yuan/month. Among them, basic salary is: ________ yuan/month |  |
| Service delivery | |  |
|  | Is the information system of your workplace connected with upper and lower-level hospitals? (1) Yes (2) No |  |
|  | In your workplace, when the same patient visits multiple times, is the service provided by the same physician? (1) Never (2) Occasionally (3) Sometimes (4) Often (5) Always |  |
|  | During non-standard working hours at the hospital (such as evenings, weekends), can patients consult about health issues via phone, WeChat, SMS, etc.? (1) Yes (2) No |  |
|  | In the past year, have you participated in organizing diabetes/hypertension self-management group activities? (1) Yes (2) No |  |
|  | In the past year, have you prescribed dietary intervention (dietary guidance, such as salt and oil restriction) to patients? (1) Yes (2) No |  |
|  | In the past year, have you prescribed dietary intervention (dietary guidance, such as salt and oil restriction) to family members of patients? (1) Yes (2) No |  |
|  | In the past year, have you prescribed exercise intervention (exercise recommendations, such as daily steps) to patients? (1) Yes (2) No |  |
|  | In the past year, have you prescribed exercise intervention (exercise recommendations, such as daily steps) to family members of patients? (1) Yes (2) No |  |
|  | In the past year, have you verified the medications taken by patients and discussed how to take them? (1) Yes (2) No |  |
|  | In the last three months, have you provided hypertension/diabetes follow-up services? (1) Yes (2) No (skip to 31) |  |
|  | How many times have you provided hypertension/diabetes follow-up services in the last three months? ⑴ 1 time ⑵ 2 times ⑶ 3 times ⑷ 4 times ⑸ 5 times or more |  |
|  | What form of hypertension/diabetes follow-up service did you provide most recently? (1) Home visit (2) Follow-up when patients visit medical institutions (4) Telephone follow-up (5) Online follow-up (mobile app, etc.) (6) Other |  |
|  | What were the main contents of the hypertension follow-up service you provided most recently? (1) Blood pressure measurement (2) Lifestyle guidance (3) Inquiring about the disease condition (4) Understanding medication usage (5) Other ____________________ |  |
|  | What were the main contents of the diabetes follow-up service you provided most recently? (1) Fasting blood glucose measurement (2) Lifestyle guidance (3) Inquiring about the disease condition (4) Understanding medication usage (5) Other ____________________ |  |
|  | During the last follow-up, did you provide health guidance to other members of the patient's family? (1) Yes (2) No |  |
|  | Do you ask patients' family members about their family medical history or other health issues? (1) Never (2) Occasionally (3) Sometimes (4) Often (5) Always |  |
|  | When developing treatment plans for patients and their families, do you ask for the patients' thoughts and opinions? (1) Never (2) Occasionally (3) Sometimes (4) Often (5) Always |  |
|  | If friends or relatives need treatment at your workplace, are you satisfied with the quality of medical and health services provided by your workplace? (1) Extremely satisfied (2) Very satisfied (3) Somewhat satisfied (4) Not very satisfied (5) Not at all satisfied |  |
|  | Are you satisfied with the quality of medical and health services you provide to patients? (1) Extremely satisfied (2) Very satisfied (3) Somewhat satisfied (4) Not very satisfied (5) Not at all satisfied |  |
| ***(for village doctors, doctors in township health centers and county hospitals)* The following questions are about your most recent patient visit:** | |  |
|  | (doctors in county hospitals) Do you usually understand the treatment situation of patients in township health centers? (1) Never (2) Occasionally (3) Sometimes (4) Often (5) Always |  |
|  | (doctors in township health centers) Do you usually understand the treatment situation of patients in county hospitals? (1) Never (2) Occasionally (3) Sometimes (4) Often (5) Always |  |
|  | (village doctors) Do you usually understand the treatment situation of patients in township health centers and county hospitals? (1) Never (2) Occasionally (3) Sometimes (4) Often (5) Always |  |
|  | (doctors in township health centers and county hospitals) Do you understand the information in the patient's health record (or hypertension/diabetes follow-up)? (1) Extremely understand (2) Very understand (3) Quite understand (4) Not very understand (5) Not understand at all |  |
|  | Your perception of your attitude? (1) Very good (2) Good (3) Average (4) Not good (5) Very bad |  |
|  | Do you believe you respect patient privacy? (1) Very good (2) Good (3) Average (4) Not good (5) Very bad |  |
|  | Do you think you give patients sufficient time to ask questions about their condition or treatment? (1) Very good (2) Good (3) Average (4) Not good (5) Very bad |  |
|  | How clear do you think your explanation of the treatment plan is? (1) Very good (2) Good (3) Average (4) Not good (5) Very bad |  |
|  | How well do you think you listen to the patient's condition? (1) Very good (2) Good (3) Average (4) Not good (5) Very bad |  |
| ***(for village doctors, doctors in township health centers and county hospitals 44-48)* The following questions are about your most recent patient referal:** | |  |
|  | Who decides whether a patient should be referred? (1) Me (2) Patient (3) Joint decision |  |
|  | Do you discuss and recommend relevant departments with the patient? (1) Yes (2) No |  |
|  | Do you provide medical records and other relevant materials for the county hospital doctors to refer to? (1) Yes (2) No |  |
|  | Do you or other staff at your medical institution help patients make appointments for registration? (1) Yes (2) No |  |
|  | What do you consider the most important aspect of referral? (Choose one) (1) Discussing and recommending relevant departments and doctors (2) Assisting with appointment scheduling (3) Stating the reasons for referral (4) Monitoring the treatment at the county hospital (5) Other: _________ |  |
| **Satisfaction** | | |
|  | How satisfied are you with the equipment and instruments used for clinical diagnosis in our hospital in meeting your daily diagnostic needs? (1) Completely satisfied (2) Very satisfied (3) Moderately satisfied (4) Not very satisfied (5) Not satisfied at all |  |
|  | How satisfied are you with the variety of medications available in our hospital in meeting your daily diagnostic needs? (1) Completely satisfied (2) Very satisfied (3) Moderately satisfied (4) Not very satisfied (5) Not satisfied at all |  |
|  | To what extent can you utilize the support of superior hospitals or external institutions' equipment and devices for patient diagnosis and treatment? (1) Fully utilized (2) Very utilized (3) Moderately utilized (4) Not very utilized (5) Not utilized at all |  |
|  | Have you joined the diabetes and hypertension health management team? (1) Yes (2) No (Skip to 55) |  |
|  | Are you satisfied with the incentive mechanism/performance scheme of the diabetes and hypertension health management team? (1) Extremely satisfied (2) Very satisfied (3) Moderately satisfied (4) Not very satisfied (5) Not satisfied at all |  |
|  | Are you satisfied with the division of labor in the diabetes and hypertension health management team? (1) Extremely satisfied (2) Very satisfied (3) Moderately satisfied (4) Not very satisfied (5) Not satisfied at all |  |
|  | Overall, are you satisfied with your current job? (1) Extremely satisfied (2) Very satisfied (3) Moderately satisfied (4) Not very satisfied (5) Not satisfied at all |  |
|  | Are you satisfied with the working conditions and equipment configuration in the unit? (1) Extremely satisfied (2) Very satisfied (3) Moderately satisfied (4) Not very satisfied (5) Not satisfied at all |  |
|  | Are you satisfied with the promotion and career development prospects in your work? (1) Extremely satisfied (2) Very satisfied (3) Moderately satisfied (4) Not very satisfied (5) Not satisfied at all |  |
|  | Are you satisfied with the management status of the unit? (1) Extremely satisfied (2) Very satisfied (3) Moderately satisfied (4) Not very satisfied (5) Not satisfied at all |  |
|  | Are you satisfied with the welfare benefits you receive at work? (1) Extremely satisfied (2) Very satisfied (3) Moderately satisfied (4) Not very satisfied (5) Not satisfied at all |  |
|  | Are you satisfied with your income level? (1) Extremely satisfied (2) Very satisfied (3) Moderately satisfied (4) Not very satisfied (5) Not satisfied at all |  |
| **Vignettes *(for village doctors, doctors in township health centers and county hospitals)*** | | |
|  | A patient (male, 40 years old), a farmer, presented with symptoms of polyphagia, polydipsia, and weight loss for 2 months. Two months ago, the patient experienced an unexplained increase in appetite, from 450g per day to 550g per day, reaching a maximum of 800g, while his weight gradually decreased. Within 2 months, he lost more than 3kg in weight. He also experienced increased thirst, polyuria, and had been taking oral Chinese medicine locally for a month with no obvious improvement before seeking further diagnosis and treatment at our hospital. Bowel and bladder movements were normal after illness, and sleep quality was fair.  Physical examination revealed a body temperature of 36°C, pulse rate of 80 beats per minute, and blood pressure of 120/80mmHg. The skin showed no jaundice, lymph nodes were not enlarged, pupils were equal and round, thyroid gland was non-palpable, heart and lungs were normal, abdomen was soft, and the liver and spleen were not palpable. There was no edema in both lower limbs, and tendon reflexes were normal. The Babinski sign was negative. Laboratory tests showed Hb 120g/L, WBC 7.6×10^9/L, PLT 267×10^9/L, and urinalysis revealed negative urine protein and urine glucose (+++). Fasting blood glucose was 10.78mmol/L.  **Based on the patient's medical history and physical examination results, what is your diagnosis?**  **Please make a treatment and management plan:** | |
|  | A 54-year-old male, diagnosed with hypertension during a community health survey 2 years ago, has a regular lifestyle, consumes a light diet, occasionally smokes, and previously took antihypertensive medication for 1 year. He stopped taking medication on his own after his blood pressure returned to normal. He sought medical attention recently due to elevated blood pressure and dizziness. The most likely factor causing his blood pressure fluctuation is:  (1) Emotional stress (2) Smoking (3) High-sodium diet (4) Poor medication adherence (5) Lack of exercise |  |
|  | A 58-year-old male with a history of hypertension for 7 years, with a highest blood pressure of 155/95 mmHg. He has previously been treated with Losartan and Hydrochlorothiazide, but his blood pressure has been poorly controlled. Two months ago, he started taking Amlodipine in addition to his regular medication, but experienced blood pressure fluctuations along with facial flushing and lower limb edema, so he stopped taking it and came for consultation. He has a 10-year history of smoking. His current blood pressure is 150/95 mmHg, with no murmurs heard in the auscultation areas of the valves, and a heart rate of 76 beats per minute. His blood lipids show LDL-C: 3.26 mmol/L, TG: 1.62 mmol/L.  **Based on the patient's medical history and physical examination results, what is your diagnosis?**  **Please make a treatment and management plan:** | |
|  | Middle-aged female, with a history of diabetes for many years, currently controlling her diet and taking metformin for treatment. Diabetes is well controlled. Recently developed high fever and cough after catching a cold, X-ray confirmed pneumonia. Urine glucose (+++). Admitted for treatment, apart from routine pneumonia management, how should diabetes treatment be adjusted?  (1) Strengthen dietary control, continue with metformin. (2) Increase the dose of metformin. (3) Switch to acarbose. (4) Metformin + acarbose. (5) Switch to insulin. |  |

**References**

1. Macarayan EK, Gage AD, Doubova SV, et al. Assessment of quality of primary care with facility surveys: a descriptive analysis in ten low-income and middle-income countries. *Lancet Glob Health*. 2018;6(11):e1176-e1185.

2. Veillard J, Cowling K, Bitton A, et al. Better Measurement for Performance Improvement in Low- and Middle-Income Countries: The Primary Health Care Performance Initiative (PHCPI) Experience of Conceptual Framework Development and Indicator Selection. *Milbank Q*. Dec 2017;95(4):836-883. doi:10.1111/1468-0009.12301
